# Angiotensin II Increases Endoplasmic Reticulum Stress in Adipose Tissue and Adipocytes

**DOI:** 10.1038/s41598-019-44834-8

**Published:** 2019-06-11

**Authors:** Kalhara R. Menikdiwela, Latha Ramalingam, London Allen, Shane Scoggin, Nishan S. Kalupahana, Naima Moustaid-Moussa

**Affiliations:** 1 Department of Nutritional Sciences, Texas Tech University, Lubbock, Texas USA; 2 Obesity Research Institute, Texas Tech University, Lubbock, Texas USA; 3 Department of Physiology, Faculty of Medicine, University of Peradeniya, Peradeniya, Sri Lanka

**Keywords:** Obesity, Obesity

## Abstract

The Renin Angiotensin System (RAS), a key regulator of blood pressure has been linked to metabolic disorders. We have previously reported that adipose overexpression of angiotensinogen in mice (Agt-Tg) induces obesity, in part mediated by adipose tissue inflammation, through yet unidentified mechanisms. Hence, we hypothesize that adipose tissue enrichment of angiotensinogen leads to activation of inflammatory cascades and endoplasmic reticulum (ER) stress, thereby, contributing to obesity. We used wild type (Wt), Agt-Tg and Agt-knockout (KO) mice along with 3T3-L1 and human adipocytes treated with RAS, ER stress and inflammation inhibitors. ER stress and pro-inflammation markers were significantly higher in Agt-Tg compared to Wt mice and captopril significantly reduced their expression. Furthermore, *in vitro* treatment with Ang II significantly induced ER stress and inflammation, whereas angiotensin II receptor inhibitor, telmisartan reduced RAS effects. Moreover, miR-30 family had significantly lower expression in Agt-Tg group. MiR-708-5p and -143-3p were upregulated when RAS was overexpressed, and RAS antagonists reduced miR-143-3p and -708-5p in both mouse adipose tissue and adipocytes. Activation of RAS by Ang II treatment, increased inflammation and ER stress in adipocytes mainly via AT1 receptor, possibly mediated by miR-30 family, -708-5p and/or -143-3p. Hence, RAS and mediating microRNAs could be used as potential targets to reduce RAS induced obesity and related comorbid diseases.

## Introduction

Obesity is defined as excessive fat accumulation which leads to higher risk of associated co-morbidities including diabetes, cancer, and cardiovascular disorders. Excess fat accumulation leads to adipose tissue dysfunction and is associated with chronic low-grade inflammation^1,2^. Adipose tissue dysregulation in turn promotes macrophage infiltration, and together they increase secretion of pro-inflammatory cytokines resulting in a vicious cycle leading to obesity associated inflammation which contributes to metabolic alterations including endoplasmic reticulum (ER) stress^3^.

Dysfunction of ER leads to immature protein accumulation which activates unfolded protein response (UPR) and is associated with pathways that trigger inflammation and obesity^4,5^. Previous studies have demonstrated that activation of C/EBP homologous protein (*Chop*), an ER stress marker, upregulates pro-inflammatory cytokines in cellular systems^5–8^. Conversely, inflammatory responses are also involved in induction of UPR^9^. However, the interrelationship between these two hallmarks (ER stress and inflammation) of obesity are not very clear.

Renin angiotensin system (RAS) is one of the physiological systems classically known to regulate blood pressure and plays a pivotal role in the induction of obesity. Its components are highly expressed in various tissues including adipose, liver, kidney^10–12^. During obesity, levels of angiotensin II (Ang II) and angiotensinogen (Agt), key players of the RAS system, are elevated^13–15^. Interestingly, mice overexpressing Agt, specifically in adipose tissue, have higher expression of pro-inflammatory and lower expression of anti-inflammatory cytokines respectively^14^. RAS inhibitors (e.g. captopril; angiotensin converting enzyme; ACE inhibitor) and Agt knockout (KO) reduced levels of pro-inflammatory markers in mice, indicating that adipose tissue RAS plays an important role in obesity^14,16–18^.

*In vivo* and *in vitro* studies conducted in cardiomyocytes have also shown that Ang II induces ER stress^19^. These studies also confirm that inflammation could be reduced by inhibiting RAS using its antagonists^19,20^. However, the regulation of these processes under RAS overexpression is still ambiguous. MicroRNAs (miRNA) could be potential mediators as they are capable of post-transcriptionally regulating multiple genes. Some miRNAs that regulate ER stress include miR-30 family, -708-5p and -143-3p (according to research conducted in cardiac muscle cells, vascular smooth muscle cells, and beta cells)^21–24^. Nevertheless, miRNAs involved in RAS -associated obesity are not yet known. Additionally, the interrelationship between ER stress and inflammation under RAS activation is not completely understood. Here, we hypothesize that overproduction of Agt induces ER stress and inflammation in adipocytes, thereby, contributing to obesity and associated metabolic alterations. We identified that RAS activation in adipose tissue as well as in adipocytes treated with Ang II, induced ER stress and inflammation, and this primarily occurs via the AT1 receptor. Further, we have identified a few microRNAs (miR-30c-3p, -30a-3p, -143-3p and -708-5p) as potential regulators of RAS induced ER stress and inflammation.

## Results

First, we wanted to understand the effects of adipose-specific RAS over activation, therefore, we used adipose tissue from low fat (LF) fed mice where Agt was specifically overexpressed in the adipose tissue (Agt-Tg). These mice had an obese and insulin resistant phenotype, as we demonstrated previously^14^. Additionally, epididymal fat normalized to body weight was significantly higher in LF fed Agt-Tg mice compared to wild type (Wt) mice (Supplementary Fig. 1A). Interestingly high fat (HF) fed Agt-Tg mice with or without captopril supplementation showed no differences in epididymal fat compared to Wt mice (Supplementary Fig. 1B). Adipose specific Agt-knockout (KO) mice showed no changes in body weight or adiposity in comparison to Wt littermates^18^. However, Agt inactivation in adipose tissue reduced inflammation and improved glucose intolerance compared to Wt mice^18^. By contrast, Agt-Tg mice had significantly higher HOMA-IR values compared to Wt mice (Supplementary Fig. 2A). Furthermore, plasma triglyceride levels exhibited trending increased levels (p = 0.0732) in LF fed Agt-Tg mice compared to Wt mice (Supplementary Fig. 2B). We next conducted histological analyses of adipose tissue sections using hematoxylin and eosin (H&E) staining and assessed inflammation by immunofluorescence staining of macrophage infiltration into adipose tissue. Agt-Tg mice had larger adipocytes (Supplementary Fig. 2C), and increased crown-like structures (indicated in arrows, Supplementary Fig. 2D), compared to Wt mice, indicating adipocyte hypertrophy and macrophage infiltration in adipose tissue of Agt-Tg mice. These results confirm that Agt overexpression in adipose tissue leads to an obese insulin resistant and inflamed phenotype.

**Figure 1:**
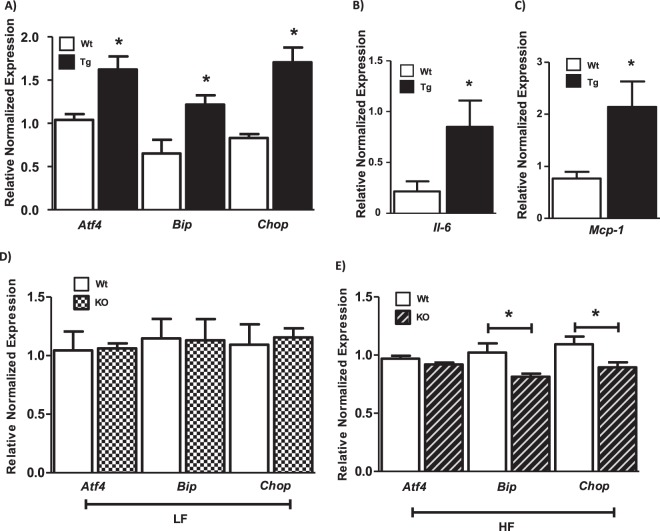
Angiotensinogen (Agt) alters endoplasmic reticulum (ER) stress & inflammation: Relative gene expression of ER stress markers normalized to *Gapdh* measured in epididymal fat pad of low fat fed male wild-type (Wt) and Agt transgenic (Tg) mice (**A**). The expression of Activating transcription factor 4 (*Atf4*), binding immunoglobulin protein (*Bip*), and C/EBP homologous protein (*Chop*) were significantly (p < 0.05) higher in Agt-Tg mice compared to control group (Wt) (**A**). Higher levels of inflammatory markers interleukin-6 (*Il-6*) and monocyte chemoattractant protein 1 (*Mcp-1*) were also observed in Agt-Tg mice compared to Wt (**B**,**C**). No changes were observed in ER stress markers in low fat fed Agt knockout mice (KO) (**D**), however, when KO mice were fed high fat die,t ER stress markers such as *Chop* and *Bip* (but not *Atf4*) showed significantly reduced expression compared to Wt group (**E**). Data is presented as mean ± SEM. (n = 6–8 each group). *p < 0.05.

Initially, we measured markers of ER stress in epididymal fat pad (VAT) of LF fed Agt-Tg mice and their Wt littermates. Activating transcription factor 4 (*Atf4*), which is induced under ER stress, was significantly higher in Agt-Tg mice compared to controls (Wt) as shown in Fig. 1A (p < 0.05; n = 6–8). Similarly, levels of other ER stress markers such as glucose regulated protein 78 (*GRP78*/*Bip*) and *Chop* were also significantly (p < 0.05) increased in Agt-Tg mice (Fig. 1A). Next, we further confirmed that RAS induction significantly activated inflammation in VAT by measuring gene levels of pro-inflammatory markers including *Il-6* and *Mcp-1* as shown in Fig. 1B & C (p < 0.05). We also, measured ER stress markers in LF fed mice where Agt was specifically knocked down in the adipose tissue. Interestingly, no differences in ER stress markers were observed (Fig. 1D). However, ER stress markers were significantly reduced in KO mice fed HF as shown by lower levels of *Bip* and *Chop* compared to Wt littermates fed HF, while no changes were observed in *Atf4* (Fig. 1E).

**Figure 2:**
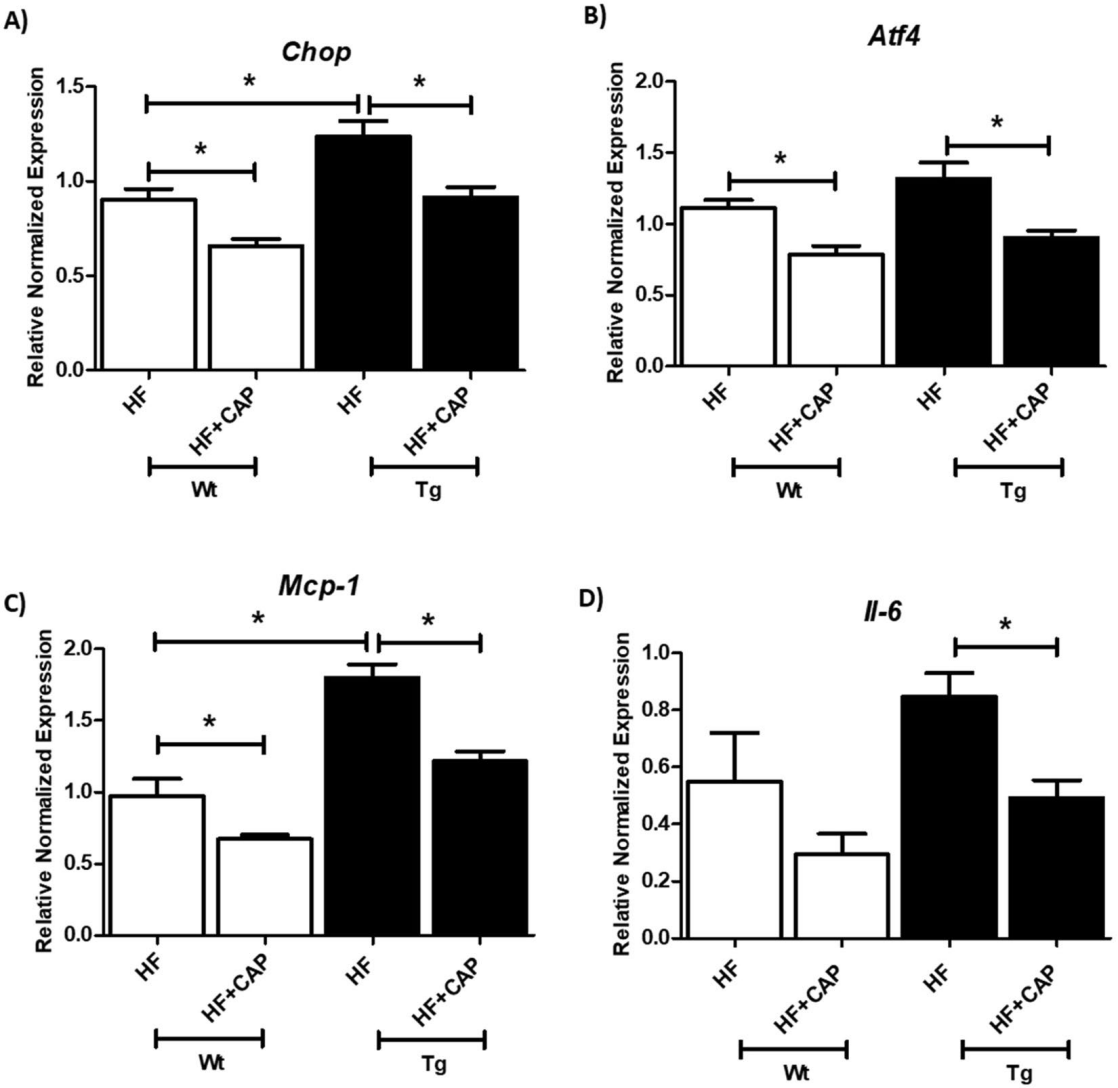
Captopril reduces ER stress and inflammation in transgenic mice overexpressing Agt in adipose tissue: Captopril attenuates high-fat diet induced ER stress and inflammation in wild-type (Wt) and Agt transgenic (Tg) mice. Half of each genotype were given Captopril (ACE inhibitor - 30 mg/L) in drinking water. Relative normalized levels of ER stress markers *Chop* (**A**), *Atf4* (**B**) and pro inflammatory markers *Mcp-1* (**C**) and *Il-6* (**D**) normalized to *Gapdh*. Data is presented as mean ± SEM. (n = 6–8 each group). *p < 0.05.

To further confirm the role of RAS pathway in ER stress, we used epididymal fat pad from Agt-Tg mice that were fed HF for 12 weeks and supplemented with or without the ACE inhibitor, captopril. Wt mice which were fed a HF diet and supplemented with captopril had significantly lower gene expression levels of *Chop* compared to Wt mice fed HF without captopril supplementation (Fig. 2A) (p < 0.05; n = 6–8). Agt-Tg mice on a HF diet had significantly (p < 0.05) higher levels of *Chop* compared to Wt mice as shown in Fig. 2A. However, when Agt-Tg mice were fed HF diet supplemented with captopril, they had reduced ER stress as shown by significantly (p < 0.05) reduced gene levels of *Chop*. Similar results were obtained for *Atf4* (Fig. 2B). We went on to determine if captopril also reduced inflammatory markers. Corroborating with ER stress, we saw higher gene levels of *Mcp-1* in the Agt-Tg mice fed HF compared to the Wt mice fed HF (Fig. 2C) (p < 0.05). Furthermore, with captopril, inflammation was reduced in Agt-Tg mice as seen with both reduced levels of *Il-6* and *Mcp1* levels in Fig. 2C,D.

**Figure 3:**
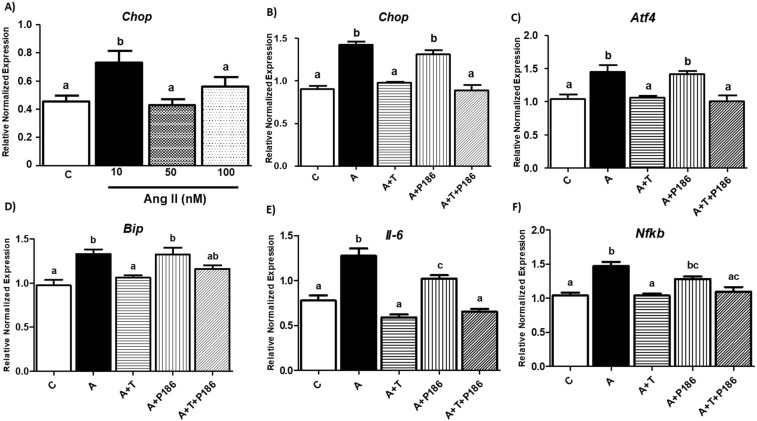
Ang II increases ER Stress and inflammation, while RAS inhibitors reduce inflammation in 3T3-L1 adipocytes *in vitro*: Adipocytes were treated with different concentration of Ang II (**A**). Differentiated adipocytes treated with media (control), Angiotensin II (Ang II), A + T (Ang II & Telmisartan), A + P-186 (Ang II & P-186), & A + T + P-186 (Ang II, Telmisartan & P-186) and ER stress as well as inflammatory markers were measured. Ang II (10 nM) treatment significantly (p < 0.05) increased ER stress markers (*Chop*, *Atf4*, *Bip*) (**B–D**) and pro inflammatory markers such as *Il-6* and nuclear factor kappa B (*Nfkb*) (**E,F**). Ang II antagonist Telmisartan significantly (p < 0.05) reduced the Ang II induced ER stress and inflammation (**B–F**). Common letters indicate no significance. Data is presented as mean ± SEM (n = 5). p < 0.05.

Epididymal adipose tissue contains adipocytes and other immune cells. Hence, to further dissect direct effects of RAS pathway induced ER stress in adipocytes, we used differentiated 3T3-L1 adipocytes. For initial experiments, mouse adipocytes were treated with 10, 50 and 100 nM doses of Ang II to identify the optimal concentration of Ang II for inducing ER stress. We identified that 10 nM Ang II induced ER stress by increased *Chop* levels compared to other doses (Fig. 3A). Moreover, concentrations above 10 nM did not induce ER stress which is consistent with previous studies where lower concentrations of Ang II were more effective in reducing fatty acid synthesis^25^. Hence, we used Ang II at 10 nM to induce ER stress for subsequent experiments.

The activity of Ang II is mediated through 2 receptors, namely angiotensin type 1 receptor (AT1) and angiotensin type 2 receptor (AT2). As RAS pathway induces ER stress, we then determined through which receptor Ang II transmits its effects. We treated mouse adipocytes with Ang II in the presence or absence of AT1 inhibitor (telmisartan) and AT2 inhibitor (P186) individually or in combination. Treatment with Ang II induced ER stress as shown by increased levels of *Chop*, *Atf4*, and *Bip* compared to control in mouse adipocytes (Figs. 3B–D) (p < 0.05; n = 5). Furthermore, combined treatment of Ang II along with telmisartan significantly (p < 0.05) reduced *Chop*, *Atf4*, *Bip* gene expression levels compared to Ang II treated cells, but P186 which blocks AT2 receptor did not reduce any of the ER stress markers compared to Ang II treated groups as shown in Fig. 3B–D. However, treatment with both inhibitors (telmisartan & P186) showed significantly (p < 0.05) lower *Atf4* and *Chop* expression levels compared to groups treated with Ang II suggesting that ER stress is primarily transmitted via the AT1 receptor (Figs. 3B–D) (p < 0.05; n = 5). Similarly, we also determined the receptor through which inflammation is mediated in the RAS pathway by measuring *Il-6* levels. Ang II increased the mRNA levels of inflammation measured by *Nfkb* (Fig. 3F) and its downstream target *Il-6* (Fig. 3E) compared to the control. Interestingly, inhibiting either of the two receptors individually or together reduced *Il-6* gene levels compared to the Ang II alone treated group (Fig. 3E) (p < 0.05). However, *Nfkb* gene levels were reduced by inhibiting AT1 and not AT2 (Fig. 3F).

**Figure 4:**
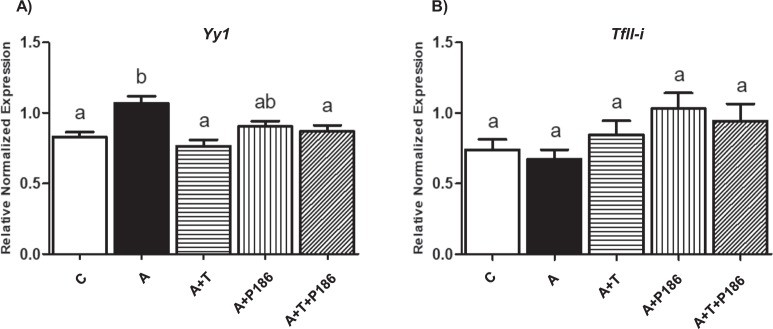
RAS possibly activates ER stress by inducing *Yy1* expression: *Yy1* (a transcription factor which regulates expression of ER stress markers) levels were significantly upregulated in Ang II treated cells compared to control group (**A**). Groups with RAS inhibitor telmisartan (A + T) and combination of telmisartan with P186 (A + B) showed significantly lower expression of *Yy1* when compared to Ang II group. However, AT2 receptor blocker P-186 (A + P186) did not significantly reduce *Yy1* expression (**A**). Interestingly, neither Ang II nor RAS antagonists changed the expression of *TfII-i* (**B**). Common letters indicate no significance. Data is presented as mean ± SEM (n = 3). p < 0.05.

Additionally, to further identify possible links between RAS and ER stress, we used Yin Yang 1 (*Yy1)* and *TfII-i* (also known as general transcription factor II-i (*Gtf2i*)), two transcription factors which regulate ER stress genes by binding to ER stress response element of GRP78/BIP^26,27^. *Yy1* expression was significantly upregulated in Ang II treated cells compared to control group, suggesting that Ang II in part increases ER stress via *Yy1* activation. Moreover, blocking Ang II binding to AT1 or both AT1 and AT2 receptors, using telmisartan without or with P186 (A + B) showed significantly lower expression of *Yy1* compared to Ang II treated cells (Fig. 4A). Similar to other ER stress markers, P186 did not change *Yy1* expression, further demonstrating that RAS induces ER stress primarily through AT1 receptor. Interestingly, neither Ang II nor RAS inhibition altered expression of *TfII-i* expression, indicating that RAS effects on ER stress may be mediated by *Yy1* but not *TfII-i* (Fig. 4B).

**Figure 5:**
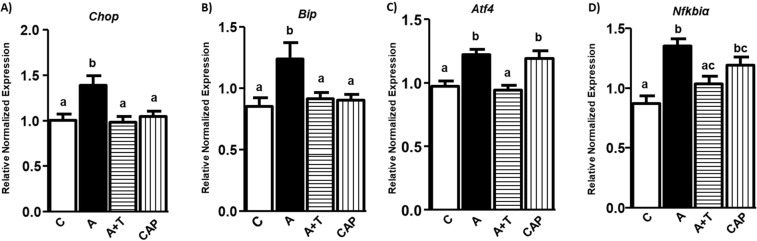
Ang II increases ER Stress and inflammation, while RAS inhibitors reduce inflammation in human mesenchymal stem cells (HMSC) *in vitro*: Differentiated HMSCs treated with media (control), Angiotensin II (Ang II (1 nM)), A + T (Ang II & Telmisartan (1 mg/ml)), or Captopril (CAP (1 mg/ml)). ER stress and inflammatory markers were measured. Ang II significantly (p < 0.05) increased ER stress markers (*Chop*, *Atf4*, *Bip*) (**A–C**) and nuclear factor kappa B inhibitor α (*Nfkbiα*) which is upregulated by the activation of *Nfkb* (**D**). Ang II antagonist Telmisartan significantly (p < 0.05) reduced the Ang II induced ER stress and inflammation (**A–D**). Common letters indicate no significance. Data is presented as mean ± SEM (n = 3). p < 0.05.

To gain further insight into potential translation of our findings to humans, we confirmed our RAS effects on ER stress data from mouse 3T3-L1 adipocytes human mesenchymal stem cells (HMSC) which were differentiated into adipocytes. Angiotensin induced ER stress in human adipocytes as shown by higher gene expression levels of *Chop*, *Bip* and *Atf4* in Fig. 5 A-C (p < 0.05; n = 3). We used only AT1 inhibitor (along with Ang II) in HMSC as we saw only its inhibition effects in clonal mouse adipocytes. Similar to 3T3-L1 adipocytes, inhibiting Ang II interaction with AT1 receptor in human adipocytes also reduced ER stress as indicated by reduction of all three markers (Fig. 5A–C). We also confirmed the effects of inflammation in human adipocytes by measuring *Nfkbiα* and found similar effects to *Nfkb* as in 3T3-L1 adipocytes (Fig. 5D) (p < 0.05). Additionally, we determined if inhibiting the RAS pathway with captopril (without Ang II stimulation) reduced ER stress. Captopril did not reduce any of the ER stress markers in comparison with control, while a rather unexpected high expression was observed in *Atf4* and *Nfkbiα*.

Captopril treatment may activate other RAS pathways (e.g. Ang1–7 activity through MAS receptor) and research evidence showed that nitric oxide (NO) production via MAS receptor could induce expression of *Atf4* in HMSC derived osteoblasts and chondrocytes^28–30^. To confirm this, mRNA levels of *Ace2* and endothelial nitric oxide synthase (eNos) were measured in HMSCs. *Ace2* expression was significantly higher in captopril treated group compared to control and Ang II treated groups, indicating that blocking the RAS pathway by captopril may increase other pathways (Ang1–7) (Supplementary Fig. 3A). However, AT1 inhibition did not alter *Ace2* in HMSC (Supplementary Fig. 3A). We also tested expression of *eNos* in these cells, however, its expression level was low to detect in these cells.

**Figure 6:**
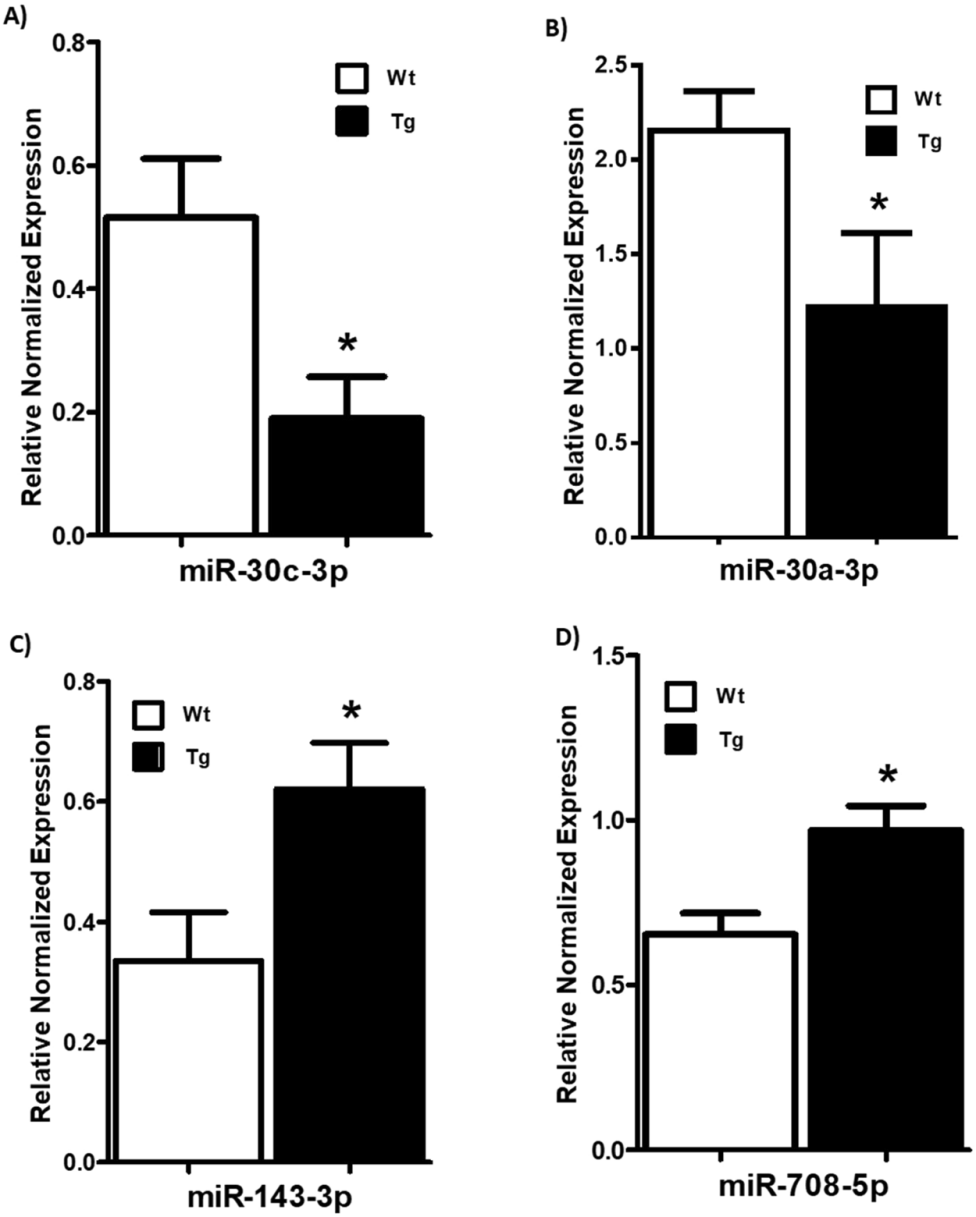
MicroRNAs levels in Agt-Tg and Wt mice fed low fat diet: Agt-Tg mice showed significantly (p < 0.05) reduced expression of miR-30 family compared to control (Wt) mice (**A,B**), whereas upregulation of miR-708-5p and miR-143-3p induced ER stress compared to Wt mice (**C,D**). Common letters indicate no significance. Data is presented as mean ± SEM (n = 6–8). *p < 0.05.

**Figure 7:**
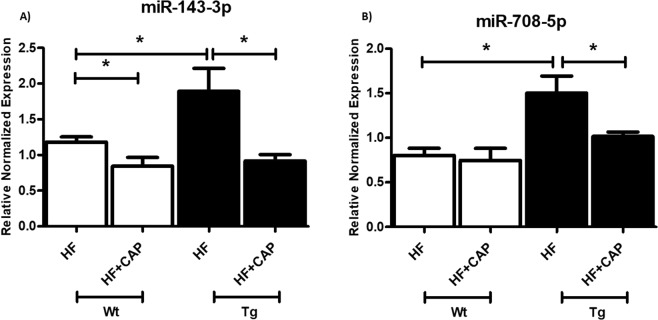
MicroRNAs levels in Agt-Tg and Wt mice fed high fat (HF) with or without captopril supplementation: Agt-Tg mice fed HF showed significantly (p < 0.05) higher levels of miR-708-5p and miR-143-3p (**A,B**) compared to Wt mice fed HF. Captopril supplementation reduced miR-143-3p in both Agt-Tg and Wt mice (**A**), whereas in miR-708-5p captopril reduced the expression significantly in Agt-Tg compared to HF group but not in Wt group. Data is presented as mean ± SEM (n = 6). *p < 0.05.

To further gain insight into RAS effects on obesity-associated ER stress and inflammation, we analyzed miRNAs reported to target genes involved in ER stress and inflammatory pathway in other tissues/cells. We tested miR-30 family which is down regulated with ER stress in cardiac muscle cells^21^. We found that in LF fed Agt-Tg mice, miR-30c-3p and miR-30a-3p levels were indeed significantly reduced in comparison to Wt mice (Figs. 6A,B) (p < 0.05; n = 6–8). Additionally, we also tested miRNAs such as miR-143-3p and miR-708-5p which are upregulated with ER stress^23,24^. As expected, we found that with RAS overexpression (Tg mice), miR-143-3p and miR-708-5p were significantly upregulated as shown in Fig. 6C,D (p < 0.05). Interestingly, miR-143-3p and miR-708-5p were also significantly higher in Agt-Tg mice fed HF compared to Wt littermates indicating probable association of these miRNA with RAS over activation (Fig. 7A,B). Further, miR-143-3p levels were reduced by captopril supplementation in both Wt and Agt-Tg mice as shown in Fig. 7A and similar reduction in miR-708-5p was observed in Agt-Tg mice with captopril but not in Wt (Fig. 7B) (p < 0.05). Lastly, miR-30 family was not altered with HF diet (data not shown).

**Figure 8:**
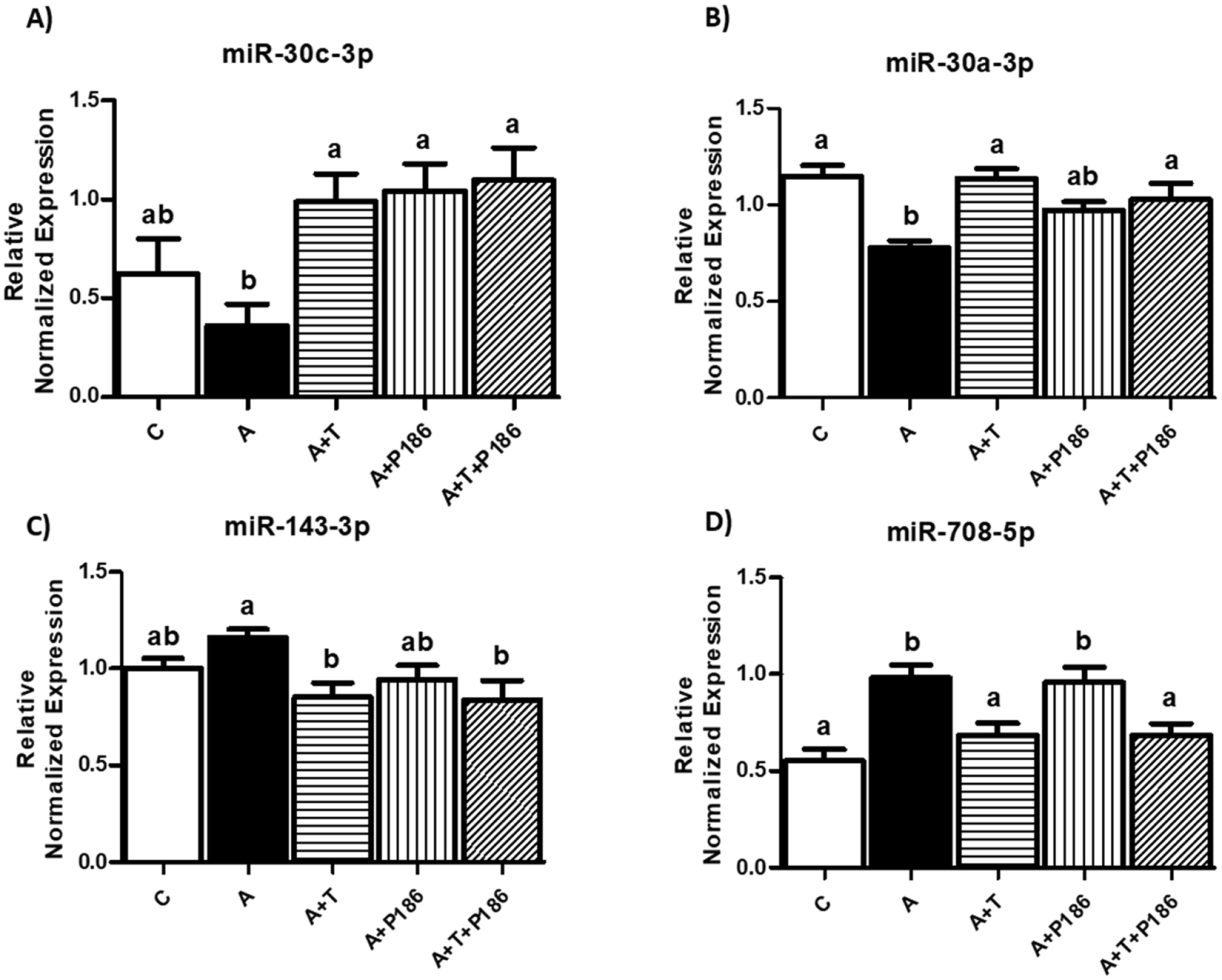
Levels of microRNAs in adipocytes treated with angiotensin II (Ang II) and the receptor inhibitors: Ang II treated 3T3-L1 adipocytes had significantly (p < 0.05) reduced expression of miR-30 family compared to control (**A,B**). Whereas Ang II treated 3T3-L1 adipocytes had induced expression of miR-708-5p and miR-143-3p compared to control groups (**C,D**). Telmisartan reversed the effects of Ang II by upregulating miR-30 family or downregulating miR-708-5p and miR-143-3p *in vitro* (**A,B**). C: Control, A: Ang II, A + T: Ang II + AT1 blocker Telmisartan, A + P-186: Ang II + AT2 blocker, A + T + P-186: Ang II + AT1 blocker Telmisartan + AT2 blocker. Common letters indicate no significance. Data is presented as mean ± SEM (n = 6–8). p < 0.05.

We further validated these miRNAs in 3T3-L1 adipocytes and found that these miRNAs were regulated in the same manner by angiotensin treatment as in adipose tissue of LF fed Agt-Tg mice. The expression of miR-708-5p was significantly upregulated in the Ang II treated group compared to control group (Fig. 8D) (p < 0.05), however, miR-143-3p expression was not significantly upregulated by Ang II treatment compared to control group (Fig. 8C). MiR-30a-3p demonstrated significantly (p < 0.05) reduced expression with Ang II treatments as observed in Agt-Tg mice (Fig. 8B) and miR-30c-3p showed reduced expression yet it was not significant (Fig. 8A). Furthermore, treatment with the RAS receptor inhibitors, increased the levels of miR-30a and c (Fig. 8A,B) while telmisartan alone or in combination with P186 reduced the levels of miR-708 and miR-143-3p as expected (p < 0.05) (Fig. 8C,D). Moreover, AT1 and AT2 receptors seem to regulate miR-30c-3p expression in a similar fashion as both the inhibitors (telmisartan and P186) have increased miR-30c-3p expression compared to Ang II group.

**Figure 9:**
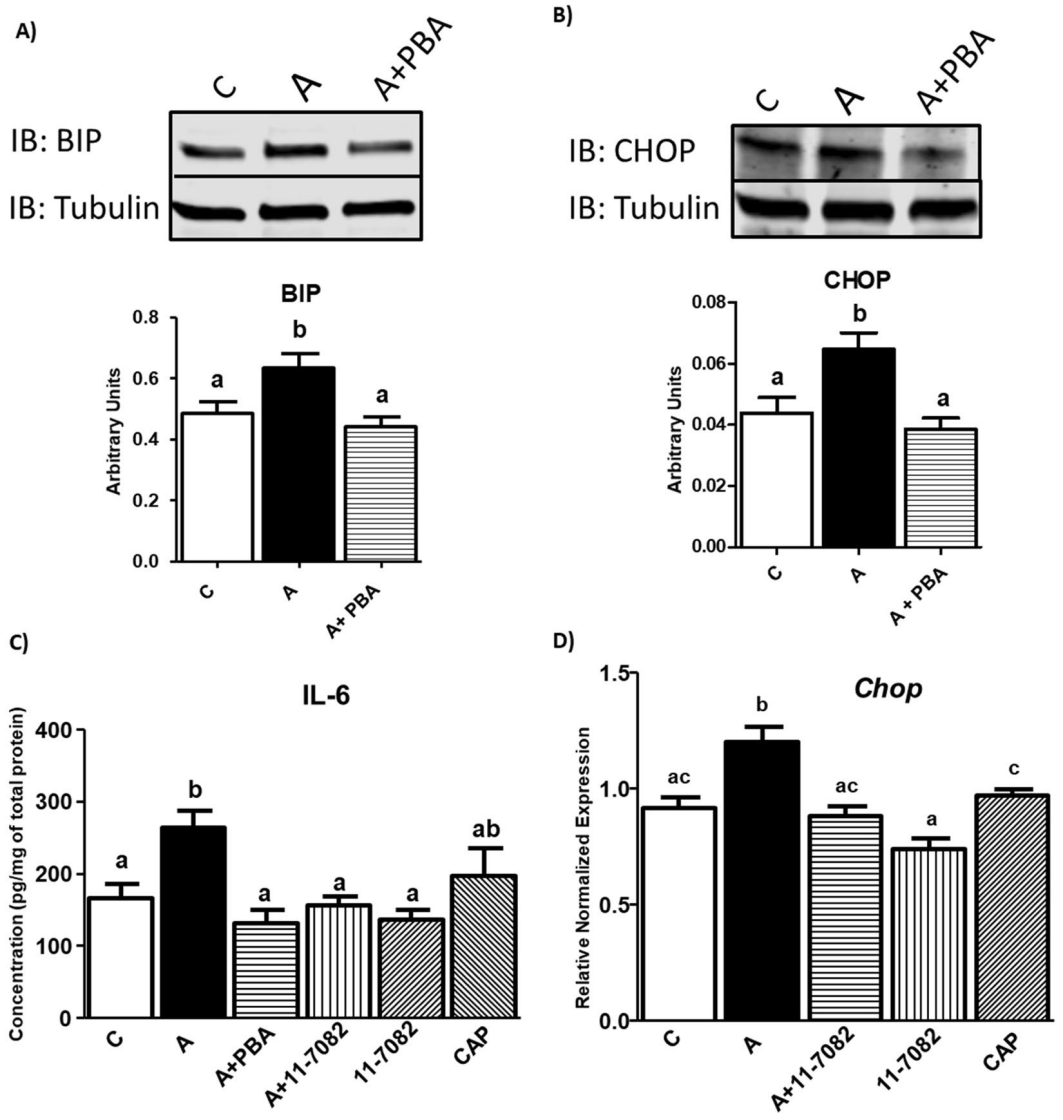
ER stress and NF-κB inhibitors (PBA and 11–7082) reduced RAS-induced inflammation and ER stress *in vitro*: Ang II increased ER stress as shown by the intensity of the GRP78/BIP and CHOP bands (**A,B**). PBA reduced the levels of GRP78 and CHOP similar to the control levels (**A,B**). Full-length blots are presented in Supplementary Figure 2A-B. Differentiated adipocytes were treated with media C (control), Angiotensin II (Ang II), NF-κB inhibitor 11-7082, ER stress inhibitor PBA (4-Phenyl Butyric acid), and ACE inhibitor Captopril. Both inhibitors (11-7082 & PBA) reduced Ang II induced inflammation assessed by IL-6 concentration (**C**). Similarly, Ang II induced ER stress is significantly (p < 0.05) reduced when inflammation was inhibited (*Chop* gene expression) (**D**). C: Control, A: Ang II, A + PBA: Ang II + 4-Phenyl Butyric acid, A + 11-7082: Ang II + NF-κB inhibitor, CAP: Captopril. Common letters indicate no significance. Data is presented as mean ± SEM (n = 3). p < 0.05.

Lastly, to further understand mechanisms by which RAS induces ER stress, we determined if reduction of RAS pathway-induced ER stress can alleviate inflammation and vice versa. For these purposes, 3T3-L1 adipocytes were treated with both inflammation and ER stress inhibitors and markers of ER stress and inflammation were measured. ER stress was inhibited with 4-phenyl butyric acid (4-PBA) along with Ang II, and ER stress was measured using GRP78 at the protein levels as we did not observe reductions with ER stress at gene levels with PBA as previously demonstrated^31^. Treating cells with Ang II increased ER stress by 20% as shown by the intensity of the GRP78/BIP band (Fig. 9A). PBA reduced the levels of GRP78 similar to the control levels indicating reducedER stress (Fig. 9A). Similar results were observed with CHOP (Gadd 153), which is another ER stress marker as shown in Fig. 9B. We next identified if inhibiting ER stress also reduced inflammation by measuring IL-6 protein levels. Interestingly, PBA treatment prevented the Ang II-mediated increase in IL-6 levels (Fig. 9C) (p < 0.05; n = 3), suggesting that Ang II-mediated inflammation is at least in part mediated via increased ER stress. We also treated cells with NF-κB inhibitor (11–7802) and confirmed its activity by measuring IL-6 levels which was significantly reduced (Fig. 9C) (p < 0.05), while captopril exhibited trending reductions in IL-6 protein levels. Next, we measured markers of ER stress after treating cells with the NF-κB inhibitor. When cells were treated with NF-κB inhibitor along with Ang II, ER stress markers were down regulated as shown by the reduced *Chop* levels (Fig. 9D) (p < 0.05), suggesting that RAS activation induces ER stress, at least in part via NF-κB pathway. These results together indicate that ER stress and inflammation are interrelated under RAS induction.

## Discussion

RAS is a well-established endocrine system and a major regulator of blood pressure and fluid balance. Over the past couple of decades, the role of RAS has expanded to numerous other functions including regulation of adiposity, inflammation and insulin resistance. However, specific mechanisms linking RAS, especially adipose RAS to obesity are largely unknown. One possibility is the involvement of ER stress which is a physiological process commonly over- stimulated during various pathological conditions including obesity. Furthermore, elevated ER stress due to obesity is considered as an underlying factor contributing to various clinical manifestations such as diabetes, and cancer^32,33^. There could be several factors that contribute to ER stress such as hypoxia, autophagy, and hyperglycemia, however, one of the common underlying factors is higher angiotensin levels^34–36^. Hence identifying the relationship between RAS, ER stress and obesity would extend our knowledge and help develop newer therapeutic targets for obesity and its associated health complications.

In this study, we identified that over activation of RAS pathway through Agt overexpression or Ang II treatment induces ER stress and inflammation in mouse adipose tissue, clonal mouse and human adipocytes. In addition, we found that Ang II-mediated ER stress is AT1 receptor dependent in adipocytes. We demonstrate here for the first time that RAS, inflammation and ER stress are closely intertwined, and Ang II induces ER stress activating transcription factor *YY1* and NF-κB pathway. Additionally, for the first time we report that miR-30 family, -143-3p and -708-5p have potential regulatory roles and are associated with adipose ER stress when RAS is activated. These findings further add to our and other previously reported research linking RAS to obesity^14^.

UPR is a critical mediator of ER stress, when upregulated activates three canonical pathways including Inositol Requiring Enzyme 1 (IRE1), Activating Transcription Factor 6 (ATF6), and PERK^4,5,37^. However, we specifically focused on the PERK-ATF4 pathway branch of ER stress since it has a major role in lipogenesis and links to inflammation. Furthermore, studies in adipocytes have indicated that either overexpressing or inhibiting IRE1 alpha does not alter adipogenesis^38^. Thus, we measured (at both mRNA and protein levels) a central regulator of the three pathways of ER stress, *Bip*, and *Chop* whose transcription is also regulated by all three ER stress pathways^39^. Our results demonstrated that, when Agt is specifically overexpressed in mouse adipose tissue, it significantly upregulated the expression of ER stress markers such as *Bip*, *Chop* and *Atf4* confirming the role of RAS in the generation of UPRs. Similar results were obtained from clonal mouse adipocytes and human adipocytes, further confirming the above findings. In addition, we have shown that the use of RAS antagonists would be a potential therapeutic approach to alleviate RAS associated metabolic alterations including ER stress and inflammation. Interestingly, effects of PBA on *Chop* was observed only at protein and not at gene levels as observed by others^31^. This could be due to post translational modification of the protein and/or role of miRNAs due to their involvement in post transcriptional modifications. These results together confirm our hypothesis that over activation of RAS leads to obesity via ER stress pathway.

Besides ER stress, chronic inflammation also plays a critical role in the pathogenesis of obesity-associated metabolic complications. Mice over-expressing Agt had higher levels of pro-inflammatory cytokines such as IL-6 in adipose tissue and vascular system^14,40^. Based on this data, we sought to validate the role of RAS in inflammation using clonal 3T3-L1 adipocytes and HMSC and found similar effects as observed in mice^14^. Inflammation is induced by many pathways in adipose tissue including the NF-κB, JNK/ERK and STAT. However, our focus was on NF-κB pathway as it is the primary inflammatory pathway induced by RAS^41–45^. While other pathways such as JNK/MAPK and STAT could also play a role in inflammation, these additional pathways need further studies and are beyond the scope of this study.

We observed that ER stress was induced when the RAS pathway was overactivated in adipose tissue. By contrast, blocking the RAS pathway (angiotensinogen knockout mice, Agt KO) did not reduce ER stress in LF-fed mice which might be possibly due to already low ER stress and/or persistent basal systemic RAS activity present in these mice. However, in KO mice fed HF diets, ER stress was significantly reduced compared to Wt mice suggesting that adipose RAS plays a pivotal role regulating ER stress during diet-induced obesity. Additionally, we did not observe reductions in ER stress when cells were treated with captopril (enzyme inhibiting the conversion of Ang I to Ang II) especially in HMSC, rather unexpected higher expression was observed in *Atf4* and *Nfkbiα*. There are few possible avenues through which the *Atf4* and *Nfkbiα* activation could occur. First, captopril treatment upregulates the ACE2/Ang 1–7 axis through the MAS receptor, previously shown to increase *Atf4* through nitric oxide (NO) in human chondrocytes and osteoblasts^28–30^. This is consistent with our data (Supplementary Fig. 3A) demonstrating significantly higher *Ace2* expression in captopril treated group compared to control and Ang II treated groups. We also tested *eNos* but it had low expression in HMSCs. These data suggest that increased *Atf4* expression could possibly be mediated by the activation of Ang 1–7. Second possibility for increase in *Atf4* and *Nfkbiα* is based on studies that have shown captopril to have off targets^46^. Third, higher *Nfkbiα* expression with captopril corroborates with other studies where they observed increased pro-inflammatory cytokines with captopril treatments^47,48^. Lastly, although we use captopril to inhibit Ang I conversion to Ang II, angiotensinogen could still be converted to Ang II by other chymase controlled pathways and the amount of Ang II produced by these pathways could be high enough to activate *Atf4* but not *Chop* or *Bip* as different doses of Ang II may stimulate genes differently. Future studies are warranted to understand the possible role of Ang 1–7/ MAS receptor and its possible involvement in generating these unexpected results with captopril in cell culture experiments. However, when Ang II interaction with its receptor AT1 was disrupted by antagonists (telmisartan), Ang II induced ER stress and inflammation were significantly reduced in both clonal mouse and human adipocytes. These results emphasize the importance of identifying tissue specific modulators (such as miRNAs) which could be used as probable targets to prevent Ang II mediated deleterious effects without targeting systemic RAS.

We next studied several miRNAs, potential modulators of ER stress in obesity. Downregulation of miR-30a induces ER stress in cardiac and smooth muscle cells as well as in cancer cells which are consistent with our results^21,22^. Furthermore, miR-30a has a role in inflammation in adipose tissue and islets indicating its relevance in obesity^49^. Similarly, miR-30c, belonging to miR-30 family, alters ER stress marker, XBP1. In this study, we found that both miR-30a and c were downregulated in the mice overexpressing Agt, along with increased ER stress markers. Other studies (cardiac muscle and vascular smooth muscle cells) have demonstrated that inhibition of miR-30a increased *chop* and *bip* levels revealing their potential relationship^21^. We found miR-708-5p was significantly upregulated (both *in vivo* and *in vitro* experiments) with ER stress which is consistent with studies in beta cells, where it is elevated in obesity^23,50^. Furthermore, this miRNA was identified as an indirect target of *Chop* where, overexpression of miR-708-5p could induce apoptosis through *Chop* mediated ER stress pathways^51,52^. MiR-143-3p regulates ER stress where over expression of miR-143-3p indicates cell stress^24^. Corroborating with these results, we reported that RAS over activation significantly upregulates miR-143-3p expression in adipocytes, indicating a potential role of miR-143-3p in RAS induced ER stress. Additionally, the expression of miR-143-3p and miR-708-5p was reduced with RAS antagonists (captopril, telmisartan) in both *in vivo* and *in vitro* experiments further confirming the possible association of these miRNAs with RAS. The regulation of ER stress genes and miRNAs was mediated through AT1 receptor, except for miR-30c-3p, which was mediated via both AT1 and AT2 receptors indicating the possibility of off target effects of this miRNA. AT1 and AT2 receptors in most cases have opposing functions related to hypertension, however, they exert similar functions in processes like adipose metabolism^53,54^. Hence, miRNAs could have same effect on both receptors and receptors may exert similar functions as shown in other studies. For example, studies by our lab showed that AT2 knockouts are resistant to diet-induced obesity^55^, just as AT1 knockouts are resistant to high fat diet-induced obesity^54^. Although, an ambiguity is seen, in the way AT2 receptor is involved in the regulation of miRNAs, a steady and constant regulatory pattern was observed in AT1 mediated miRNA regulation, further confirming the suitability of AT1 receptor as a potential therapeutic target of RAS mediated metabolic disorders.

In this study, we identified that inflammation and ER stress are interlinked when Agt is overexpressed. The relationship between ER stress and inflammation is not surprising given their role in metabolic alterations, but their relationship remains paradoxical in terms of the RAS pathway. Interestingly, activation of all three pathways of the UPR could induce inflammatory pathways including NF-κB^56^. Furthermore, inflammatory mediators induce ER stress directly or indirectly. However, whether such an interrelationship exists in all tissue systems is not clear. Potential mechanisms by which induction of RAS pathway may contribute to metabolic dysfunction in obesity through ER stress include (a) Over activation of the RAS pathway induces ER stress which could upregulate levels of its resident protein SREBP;^57^ This would further induce its downstream targets including SREBP1c, which in part activates fatty acid synthesis leading to lipid accumulation and obesity^58^. (b) Excess nutrients are usually sequestrated in the adipose tissue^59^, but beyond its storage capacity, it stresses the adipocytes leading to metabolic complications including ER stress, inflammation, hypoxia and altered adipokine signaling^57^. (c) Early stage disruption of adipocyte function could promote ER stress and obesity. This is critical as the ER is the site of protein synthesis and triglyceride formation^57,60^. However, whether ER stress afflicts lipid formation and adipogenesis needs to be further addressed. (d) In addition, ER stress could induce obesity by activating autophagic genes which then ameliorates adipogenesis^61,62^. Several of these processes are interconnected and these interconnections could also exist in the presence of angiotensin induction. These intertwined pathways affect other metabolic pathways disrupting the metabolic homeostasis of the system.

Hence, to identify mechanisms behind one of the metabolic pathways such as RAS, we used inhibitors to block both Ang II receptors. Our study identified that blocking AT1 inhibited ER stress while we observed no effects blocking AT2. Additionally, blocking both the receptors had similar effects as blocking AT1 indicating that ER stress is primarily transmitted through AT1 receptor. This is in agreement with previous research which has shown all deleterious effects of RAS to be primarily transmitted via the AT1 receptor^63^. Furthermore, recent evidence shows that blocking the AT1 receptor induces browning of white adipose tissue which is beneficial for preventing obesity^64^. Additionally, we showed that RAS induces ER stress via AT1 receptor by activating transcription factor *Yy1*. Transcription factors such as *Yy1* and *TfII-i* activates *Grp78/Bip* expression by binding to ER stress responsive element in order to induce UPRs^26,27^. Interestingly, only *Yy1* expression was upregulated by RAS over activation but no Ang II affect was observed on *TfII-i* expression. In addition, suppression of Ang II function via AT2 antagonist (P186), didn’t affect *Yy1* expression, as observed in ER stress markers whereas AT1 blocker was able to completely reverse RAS induced *Yy1* expression. Wang, H *et al*. have previously demonstrated that NF-κB regulates YY1 expression at the transcriptional level, by direct binding of the p50/p65 heterodimer complex to the YY1 promoter^65^. Taken together, these evidences further confirm our finding that RAS increased ER stress in part via NF-κB-YY1 axis through AT1 receptor. Nevertheless, future studies are needed to further dissect the mechanisms linking RAS to YY1 expression, and further validate the role of Ang II- regulated miRNAs in obesity-linked ER stress, all of which are worth investigation but beyond the scope of this manuscript.

## Methods

### Cell culture

Mouse 3T3-L1 pre-adipocytes, purchased from American Type Culture Collection (ATCC) (Manassas, VA, USA), and human mesenchymal stem cells (HMSC), purchased from LONZA (Allendale, NJ, USA) were utilized in this study. Cell lines were maintained in Dulbecco’s Modified Eagle’s Medium (DMEM) supplemented with 10% fetal bovine serum and antibiotics (50 µg/ml penicillin, 100µg/ml neomycin and 50 µg/ml streptomycin; Thermo Fisher Scientific, Waltham, MA USA). 3T3-L1 pre-adipocytes were differentiated to mature adipocytes with growth media supplemented with 0.5 mM Methyl isobutyl xanthine, 0.25 μM Dexamethasone (Sigma-Aldrich, St. Louis, MO, USA) and 10 nM insulin for 3 days. After 3 days in differentiation media, cells were placed in media containing DMEM, 10% FBS, and 1µg/ml insulin for 3 days. Following which, adipocytes were maintained in growth media for 5 days for treatments. HMSC were differentiated as previously published^66^. Differentiated 3T3-L1 adipocytes as well as HMSCs were treated with various doses of Ang II (Sigma-Aldrich, St. Louis, MO, USA) for 24 hours and 48 hours respectively. For other experiments, various inhibitors such as 1 mg/ml Captopril, 1000 nM P-186 and 1 mg/ml Telmisartan, 7.5 mM PBA (Sigma-Aldrich, St. Louis, MO, USA), and 5 µM 11-7082 (Calbiochem, Burlington, MA, USA) were used. RAS was stimulated with Ang II when cells were treated with above inhibitors except for captopril. After 24 hours (3T3-L1) and 48 hours (HMSCs) of treatment, media and cells were harvested and stored in - 80 °C for further analyses.

### Gene Expression

RNA was isolated using RNeasy mini kit (Qiagen, Valencia, CA, USA). cDNA was reverse transcribed using iScript reverse transcription supermix (BioRad, Hercules, CA, USA). Gene expression levels were assessed with real-time quantitative polymerase chain reaction (RT-qPCR) using Sybr green master mix (BioRad, Hercules, CA, USA) normalized to a housekeeping gene (18 S ribosomal RNA or GAPDH). Primer list is provided in Supplementary Table 1.

### MicroRNA Expression

Total RNA was isolated using RNeasy mini kit (Qiagen, Valencia, CA, USA) and cDNA was reverse transcribed using TaqMan™ Advanced microRNA cDNA Synthesis Kit (Thermo Fisher Scientific, Waltham, MA, USA). MicroRNA expression levels were assessed with real-time quantitative polymerase chain reaction (RT-qPCR) using TaqMan™ Fast advanced master mix (Thermo Fisher Scientific, Hereford, TX, USA) and normalized to a housekeeping microRNA (miR-191-5p and miR-186-5p).

### Western blotting

Total protein concentration was measured using bradford protein assay (Bio-Rad, Hercules, CA, USA). SDS-PAGE was used for separation of whole protein lysates, following which it was transferred to Polyvinylidene Difluoride (PVDF) membranes (Millipore, Burlington, MA, USA). Blots were incubated with primary antibodies for overnight with gentle shaking at 4 °C. Primary antibodies used were mouse tubulin (Sigma-Aldrich, St. Louis, MO, USA), mouse GRP78 and Gadd 153 (Santa Cruz Biotechnology, Dallas TX, USA)^31^. The membranes were then incubated with anti-mouse or rabbit secondary antibodies for 1 hour (Jackson Immuno Research Laboratories, West Grove, PA, USA). Florescence was detected using LI-COR Odyssey machine (LI-COR Odyssey CLX, Lincoln, NE, USA).

### ELISA

IL-6 in the supernatants were measured using enzyme-linked immunosorbent assay (ELISA) kits (R&D Minneapolis, MN, USA) according to the manufacturer’s protocol.

### Mouse studies

We used low fat (LF: 10% kcal from fat) fed transgenic mice (Agt-Tg) where angiotensinogen was overexpressed specifically in the adipose tissue (using aP2 promoter) along with LF fed wildtype (Wt) mice^14^. Epididymal fat pad was collected after euthanasia (CO_2_ inhalation) from these mice at around 16 weeks of age^14^. We also used epididymal fat pad from Agt-Tg and Wt mice which were around 16 weeks and fed a high fat (HF: 45, 20, and 35% kcal from fat, protein, and carbohydrate, respectively) diet supplemented with or without captopril (30 mg/l), an angiotensin converting enzyme inhibitor, for 12 weeks to determine the effects of the RAS pathway^14^. Epididymal fat pad was also used from mice where Agt was specifically knocked down only in adipose^18^. These mice were fed a LF (10% kcal from fat) or HF diet (45% kcal) and were around 28 weeks of age when tissues were harvested. All studies were approved, and methods were performed in accordance with the University of Tennessee, institutional animal care and use committee. Experiments using adipose tissue collected from above mice were performed at Texas Tech University.

### Immunohistochemical and immunofluorescent staining

WAT sections were fixed, and paraffin embedded before stained with hematoxylin &eosin (H&E). Digital images were taken under a 20 × magnification on an EVOS FL Auto Imaging System (Thermo Fisher Scientific, Waltham, MA, USA). For macrophage infiltration, fixed WAT sections were stained with primary antibody (anti-human/mouse galectin-3; Thermo Fisher Scientific, Waltham, MA, USA) overnight at 4 °C followed by secondary antibody (CyTM3-Donkey Anti-Rat IgG; Jackson Immuno Research Laboratories, Inc., West Grove, PA,USA) staining for 2–4 hrs the following day. Images were taken at 10 × magnification using EVOS FL Auto Imaging System.

### Triglyceride assay

Triglycerides were measured using a colorimetric assay kit (Cayman chemical, Ann Arbor, MI, USA) according to the manufacturer’s protocol.

### HOMA-IR

The homeostasis model assessment for insulin resistance (HOMA-IR) was calculated by dividing the product of Fasting insulin concentration (μU/L) × fasting glucose concentration (mmol/L)/22.5.

### Statistical analyses

Results are presented as means ± SEM. One-way ANOVA was used, followed by Tukey’s post-hoc test (p < 0.05) for experiments with more than 2 groups. For data with two groups, students t test was used. Results from the qRT-PCR assays were analyzed using the CFX Manager software provided by Bio-Rad Laboratories, Inc. using 2-ΔΔCT method. All mouse experiments had 6–8 replicates and *in vitro* experiments had 3–5 replicates.

## Supplementary information

Supplementary Figures

## Data Availability

All data generated and analyzed during this study are available from the corresponding author on reasonable request.
