# Supplementary material for: Angiotensin II Increases Endoplasmic Reticulum Stress in Adipose Tissue and Adipocytes
Source: Sci Rep. 2019 Jun 11;9:8481. doi: 10.1038/s41598-019-44834-8 (PMC6560092; doi:10.1038/s41598-019-44834-8)
Supplement: Supplementary file 1 — Supplementary Figures [file 41598_2019_44834_MOESM1_ESM.pdf]

Angiotensin II Increases Endoplasmic Reticulum Stress in Adipose Tissue and Adipocytes

Kalhara R. Menikdiwela, Latha Ramalingam, London Allen, Shane Scoggin, Nishan S. Kalupahana & Naima Moustaid-Moussa

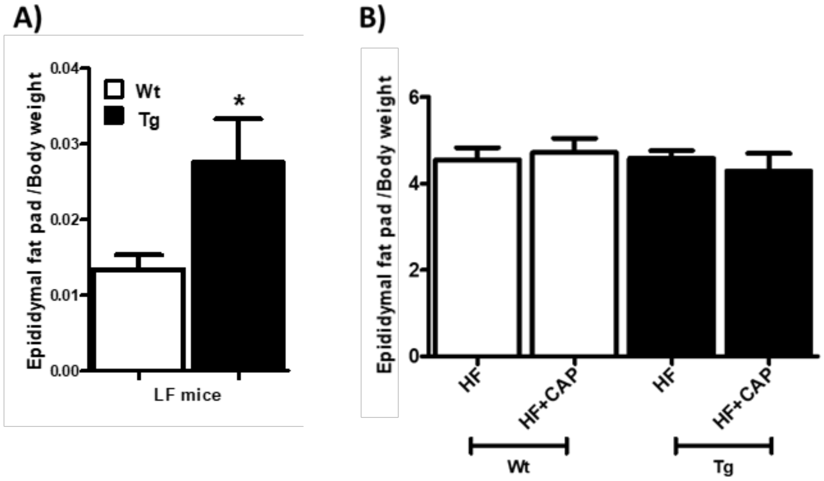

<u>Supplementary Figure 1. Epididymal fat normalized to body weight of Agt-Tg and Wt mice fed low fat and, high fat with or without captopril:</u> Epididymal fat weight normalized to body weight was significantly higher in low fat fed Agt-Tg mice compared to Wt mice (A). However, when Agt-Tg and Wt mice were fed a high fat diet, no changes were observed in normalized epididymal fad weights. Further, no differences were observed with captopril supplementation in Wt mice or Agt-Tg mice (B). Data is presented as mean ± SEM. (n=6-8 each group). * $p < 0.05$.

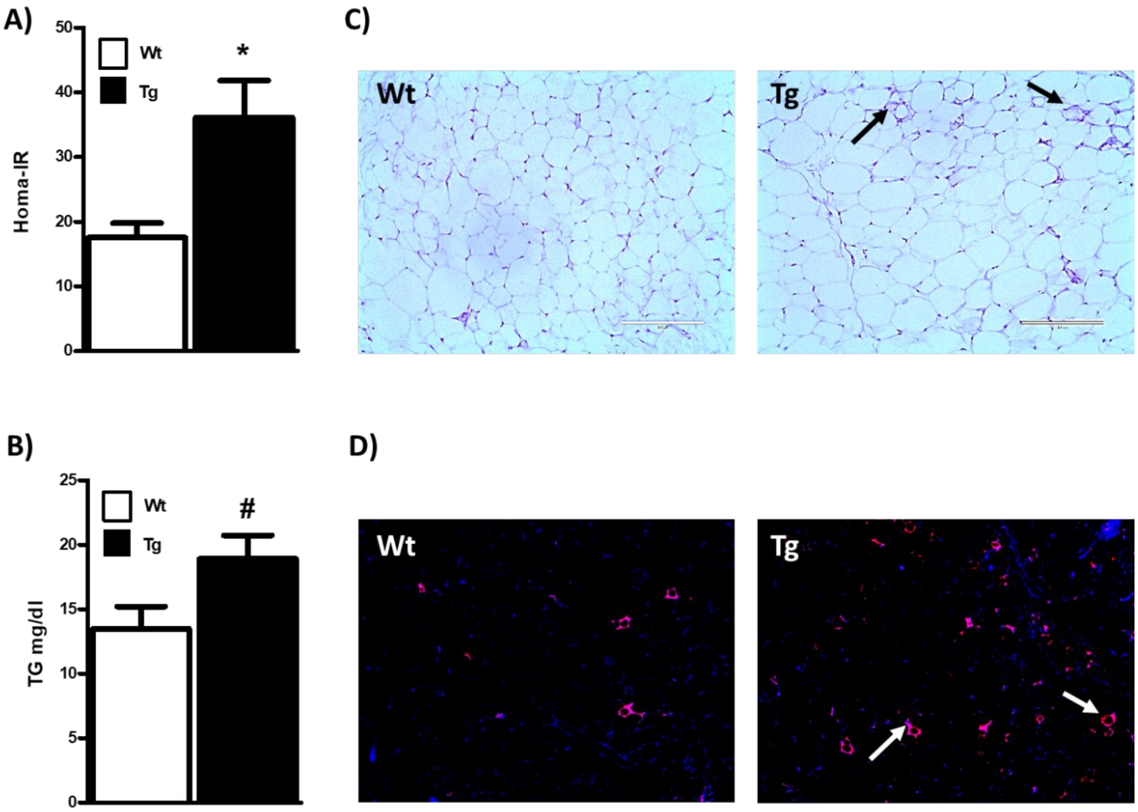

Supplementary Figure 2. Agt-Tg overexpression in adipose tissue causes obese phenotype: Agt-Tg mice showed significantly higher HOMA-IR compared Wt mice (A). Hematoxylin and eosin staining was performed on visceral fat and larger adipocytes were observed in Agt-Tg mice compared to Wt mice (C). Additionally, we observed crown-like structures (indicated in arrows), which represent macrophage infiltration around adipocytes and immunofluorescent staining (Galectin-3 pink color: macrophage marker and Cy3 blue color: nuclei; n=3) confirmed higher macrophage infiltration in Agt-Tg group compared to Wt group (D). Plasma triglyceride levels exhibited increased trends in LF fed Agt-Tg mice compared to Wt mice ($p = 0.0732$). Data is presented as mean ± SEM. (n=6-8 mice each group). * $p < 0.05$, # $p < 0.1$.

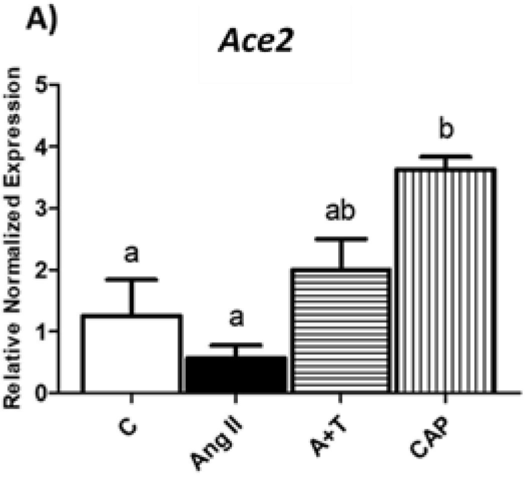

<u>Supplementary Figure 3. RAS blockage by captopril activates other RAS pathways in HMSCs:</u> Inhibition of RAS pathway by captopril activates other pathways as HMSCs showed significalty high *Ace2* expression in CAP (captopril) group (A). However, AT1 inhibition did not alter *Ace2* in HMSC. Common letters indicate no significance. Data is presented as mean ± SEM (n=3). $p < 0.05$.

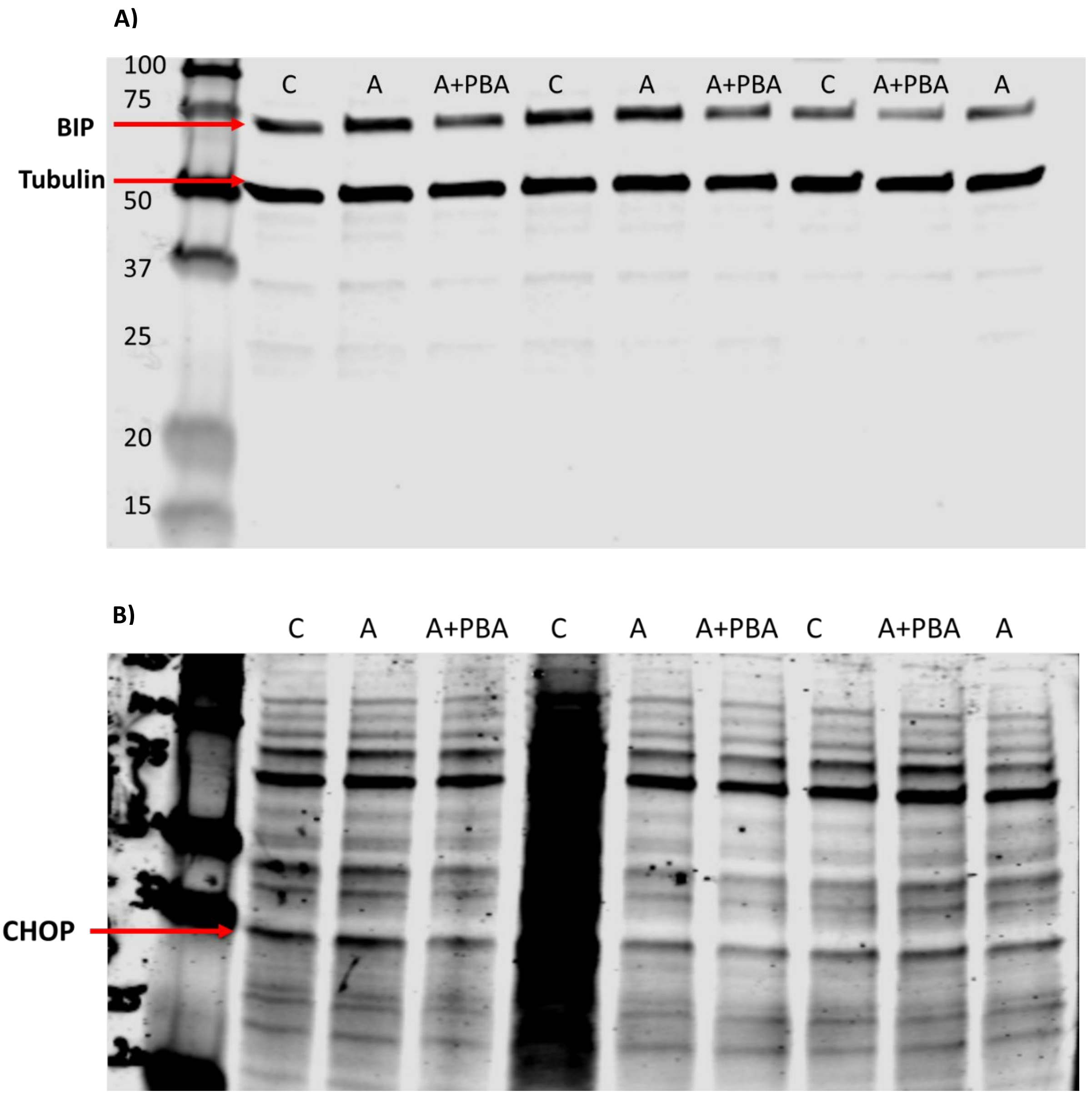

<u>Supplementary Figure 4. Full-length western blots of ER stress markers BIP and CHOP:</u>

BIP protein amount was significantly reduced when 3T3-L1 cells treated with PBA (A) and similar reduction was observed in CHOP (B)

Primer	Forward (5'-3') Sequence	Reverse (5'-3') Sequence
Mouse *Mcp1*	ACTTCTATGCCTCCTGCTCAT	GCTGCTTGTGATTCTCCTGTAG
Mouse *Atf4*	AAGCAGCAGAGTCAGGCTTTC	GGGTTCTGTCTTCCACTCCA
Mouse *Bip*	TTCAGCCAATTATCAGCAAACTCT	TTTTCTGATGTATCCTCTTCACCAGT
Mouse *Chop*	CCACCACACCTGAAAGCAGA	AGGTGAAAGGCAGGGACTCA
Mouse *Il6*	AACCGCTATGAAGTTCCTCTC	TCCTCTGTGAAGTCTCCTCTC
Mouse *NFkB*	GGTGAAGGTCGGTGTGAAC	TGAGTGGAGTCATACTGGAACA
Mouse *Yy1*	TACCTGGCATTGACCTCTC	CTATTGTTCTTGGAGCATCATCTT
Mouse *TfIIi*	TGTCCTCACTGCCATTCA	ATCCTCCACCTGCTCAAG
Mouse *Gapdh*	CTGAGTGGAGTCATACTGGAACA	GGTGAAGGTCGGTGTGAAC
Mouse *18S*	GGACAGGATTGACAGATTGATAGC	TGCCAGAGTCTCGTTCGTTA
Human *Atf4*	CCTTCACCTTCTTACAACCTCTTC	GTAGTCTGGCTTCCTATCTCCTT
Human *Bip*	CCGAGGAGGAGGACAAGAAG	AGGAGTGAAGGCGACATAGG
Human *Nfkbiα*	ACCTGGTGTCACTCCTGTT	GCTCTCCTCATCCTCACTCTC
Human *Chop*	GTCTAAGGCACTGAGCGTATC	CAGGTGTGGTGATGTATGAAGAT
Human *Ace2*	CTTCCTGGCTCCTTCTCA	TCTTCGGCTTCGTGGTTA
Human *Gapdh*	AGTCCACTGGCGTCTTCA	ATCTTGAGGCTGTTGTCATACTTC
Human *18S*	AGTCGCTCCAGGTCTTCA	GCAGAATCCACGCCAGTA

Supplementary Table 1: List of Primer
